# A realist process evaluation within the Facilitating Implementation of Research Evidence (FIRE) cluster randomised controlled international trial: an exemplar

**DOI:** 10.1186/s13012-018-0811-0

**Published:** 2018-11-16

**Authors:** Jo Rycroft-Malone, Kate Seers, Ann Catrine Eldh, Karen Cox, Nicola Crichton, Gill Harvey, Claire Hawkes, Alison Kitson, Brendan McCormack, Christel McMullan, Carole Mockford, Theo Niessen, Paul Slater, Angie Titchen, Teatske van der Zijpp, Lars Wallin

**Affiliations:** 1 Bangor Institute for Health and Medical Research, School of Healthcare Sciences, Bangor University, Bangor, UK; 2 Warwick Medical School, University of Warwick, Coventry, UK; 3 Faculty of Medicine and Health Science, Department of Nursing, Linkoping, and Department of Neurobiology, Care Sciences and Society, Division of Nursing, Karolinska Institutet, Linkopings University, Stockholm, Sweden; 4 Fontys School of People and Health Studies, Fontys University of Applied Sciences, Eindhoven, The Netherlands; 5 School of Health and Social Care, London South Bank University, 103 Borough Road, London, SE1 0AA UK; 6 Adelaide Nursing School, University of Adelaide, Adelaide, Australia; 7 College of Nursing and Health Sciences, Flinders University, Adelaide, South Australia Australia; 8 Division of Nursing, Queen Margaret University Edinburgh, Edinburgh, UK; 9 Institute of Applied Health Research, University of Birmingham, Birmingham, UK; 10 Institute of Nursing and Health Research, Ulster University, Shore Rd Belfast, Ulster, Northern Ireland; 11 Institute of Nursing and Health Research, Ulster University, Jordanstown, UK; 12 School of Education, Health and Social Studies, Dalarna University, Falun, Sweden; 13 Department of Health and Care Sciences, The Sahlgrenska Academy, University of Gothenburg, Gothenburg, Sweden

**Keywords:** Facilitation, Realist process evaluation, Implementation, PARIHS, Urinary incontinence, Context, Older people

## Abstract

**Background:**

Facilitation is a promising implementation intervention, which requires theory-informed evaluation. This paper presents an exemplar of a multi-country realist process evaluation that was embedded in the first international randomised controlled trial evaluating two types of facilitation for implementing urinary continence care recommendations. We aimed to uncover what worked (and did not work), for whom, how, why and in what circumstances during the process of implementing the facilitation interventions in practice.

**Methods:**

This realist process evaluation included theory formulation, theory testing and refining. Data were collected in 24 care home sites across four European countries. Data were collected over four time points using multiple qualitative methods: observation (372 h), interviews with staff (*n* = 357), residents (*n* = 152), next of kin (*n* = 109) and other stakeholders (*n* = 128), supplemented by facilitator activity logs. A combined inductive and deductive data analysis process focused on realist theory refinement and testing.

**Results:**

The content and approach of the two facilitation programmes prompted variable opportunities to align and realign support with the needs and expectations of facilitators and homes. This influenced their level of confidence in fulfilling the facilitator role and ability to deliver the intervention as planned. The success of intervention implementation was largely dependent on whether sites prioritised their involvement in both the study and the facilitation programme. In contexts where the study was prioritised (including release of resources) and where managers and staff support was sustained, this prompted collective engagement (as an attitude and action). Internal facilitators’ (IF) personal characteristics and abilities, including personal and formal authority, in combination with a supportive environment prompted by managers triggered the potential for learning over time. Learning over time resulted in a sense of confidence and personal growth, and enactment of the facilitation role, which resulted in practice changes.

**Conclusion:**

The scale and multi-country nature of this study provided a novel context to conduct one of the few trial embedded realist-informed process evaluations. In addition to providing an explanatory account of implementation processes, a conceptual platform for future facilitation research is presented. Finally, a realist-informed process evaluation framework is outlined, which could inform future research of this nature.

**Trial registration:**

Current controlled trials ISRCTN11598502.

## Background

The challenges of ensuring practice is informed by the best research evidence are well rehearsed. While facilitation as a role and process is shown to be a promising approach to enabling evidence-informed practice [1–3], there is a need for theory-informed evaluations of facilitation as an implementation strategy [4]. In this paper, we report on a realist-informed process evaluation, which was embedded in the first cross Europe randomised controlled trial (RCT) to evaluate two approaches to facilitating urinary incontinence recommendations in care home settings [5]. This study was novel in scale with a four cross-country setting, and as an exemplar of a realist process evaluation within a large scale international trial. The purpose was to provide a theory-driven explanation of the response to facilitation interventions as they were being implemented in practice.

Methodological guidance reinforces the importance of process evaluations in designing and evaluating complex interventions [6, 7]. Moore et al.’s process evaluation framework identifies the importance of paying attention to what is implemented, the mechanisms responsible for impact and the effect that context can have on implementation. The Standards for Reporting Implementation studies (StaRI) [8] also focus attention on the importance of reporting underpinning intervention mechanisms and the influence of the implementation context. The guidance and reporting standards both resonate with the idea of a realist-informed inquiry, which pays attention to mechanisms, context and outcomes [9, 10]. Realist inquiry is particularly helpful in providing a theory-driven explanation of how interventions and programmes, which by their nature are complex, work contingently within the context of their implementation.

There has been a lively debate about the notion of realist randomised controlled trials [11–14]. The debate centres on whether RCTs are ‘inimical to realist enquiry’ ([14], p1). Whilst RCTs and realist inquiry share some of the same language, i.e., mechanisms and contexts, there is disagreement about the meaning of those terms because of fundamentally different ontological perspectives, and a difference of opinion about whether this matters. In this research, we conducted a randomised controlled trial, which involved a process evaluation that was realist informed. In this way, we were able to remain faithful to the foundations of realist research as developed by Pawson and Tilley [9] and reap the benefits of a theory-informed approach to evaluation, whilst preserving the strengths of an RCT design.

As one of the first published examples of a realist process evaluation [15–17], we provide details about how we approached this evaluation, before presenting realist contingent explanations about how people responded to the facilitation interventions as they were being implemented. Finally, we offer a framework to help guide the conduct of future realist process evaluations.

## Methods

Our realist process evaluation enquiry, rather than identifying cause and effect relationships, aimed to uncover what worked (and did not work), for whom, how, why and in what circumstances whilst implementing and evaluating two types of facilitation interventions. See Seers et al. [5] for the trial protocol, and Seers et al. [18] for trial outcome findings.

### Design

We followed the stages of realist evaluation including theory formulation, theory testing and refining. A fundamental assumption of realist inquiry is that ‘programmes are complex interventions introduced into complex systems’ ([10]:p33) including that programmes are theories. Therefore, realist theories typically combine elements of substantive theory with stakeholders’ theories—i.e. their ideas about how programmes may work. Recognising that interventions work differently in different circumstances rather than identifying linear cause and effect relationships through secessionist logic (*x* causes *y*: often illustrated through logic models), realist enquiry is concerned with identifying the underlying generative mechanisms about how interventions work [or not]. Dalkin et al. [19] suggest that a mechanism is both the resource that an intervention provides and recipients’ reasoning and response to it. They also conceptualise mechanisms as operating on an activation continuum rather than as an ‘on-off switch’. Therefore, realist theories are those that define the underlying causal mechanisms through which outcomes occur, and the contexts in which those mechanisms are triggered or activated, which are often expressed as context (*C*) + mechanism (*M*) = outcome (*O*).

### Approach

#### Theory formulation

The trial had three intervention arms: standard dissemination, type A and type B facilitation, which were derived from the Promoting Action on Research Implementation in Health Services (PARIHS) framework [5, 18, 20, 21]. As the starting point for theory formulation, we undertook a concept mining exercise in which we identified the main elements of the interventions and PARIHS that might explain how the interventions could work in practice, and what might influence implementation. We also incorporated the geographical, policy and practice contexts of the international study into this process.

**Fig. 1:**
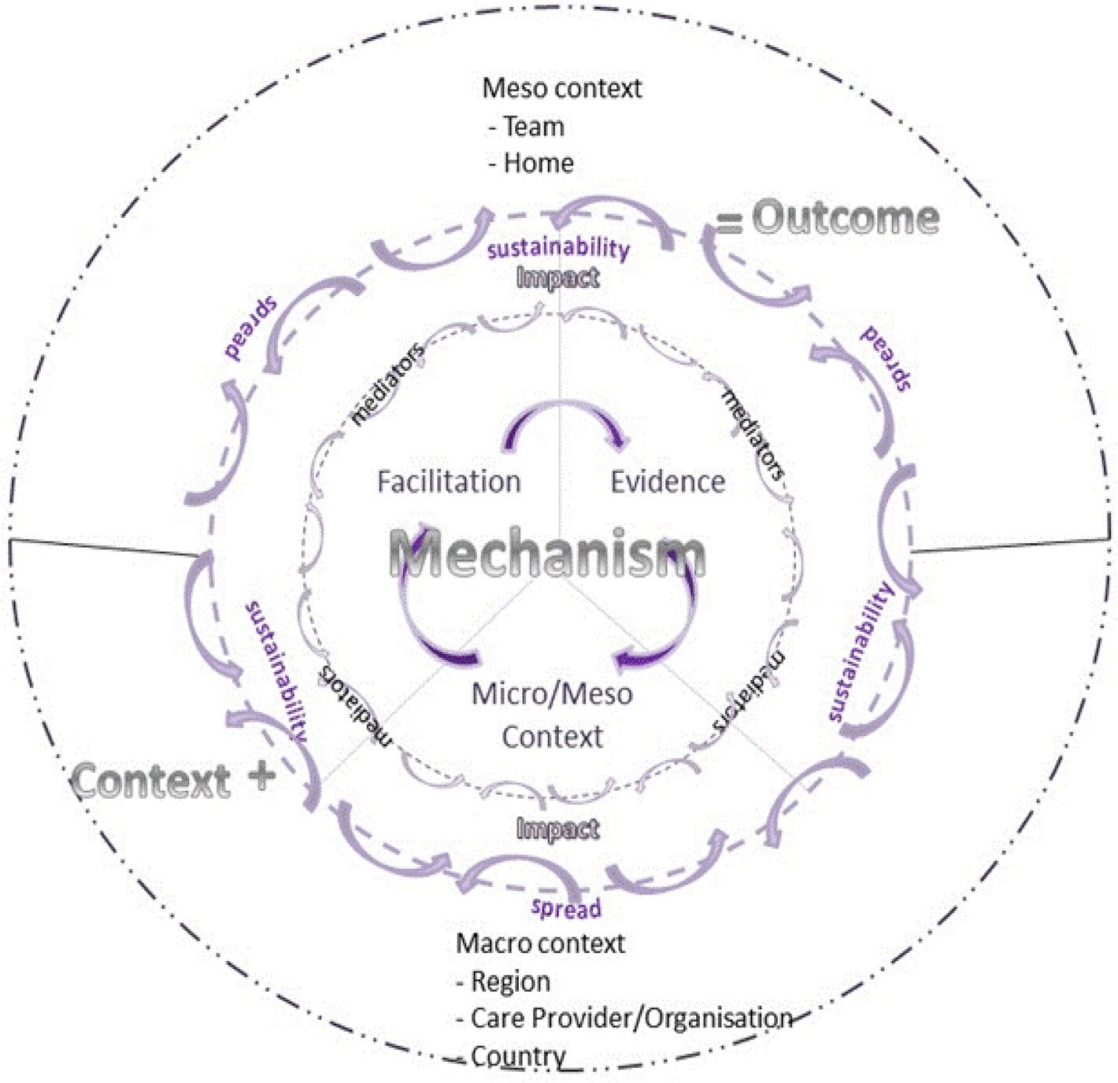
FIRE realist process evaluation framework

This process resulted in a sizable list of concepts and ideas, which we clustered into meaningful units. Consistent with the focus of realist evaluation on engaging with stakeholders, a workshop was held with 30 participants at an international knowledge utilisation colloquium. These stakeholders had a strong interest in implementation research, and some also had expertise in care home research. During the workshop, we asked participants to share ideas, i.e. personal theories, about how and why standard dissemination and facilitation interventions might work (or not) within care home settings. Following the workshop, participants and study team’s ideas were combined. These were then shared with participants at the colloquium the following year (Fig. 1 and Table 1).

**Table 1:** Framework components

Evidence—what is included in the evidence base of practice, and in the evidence base of the continence care recommendations, which has the potential to influence how care is delivered	
Practice recommendations, including their sharing and dissemination (through standard dissemination intervention)	
Practitioner experience	
Resident experience of continence care	
Local data/information about continence care/practice (including supplies)	
Context—factors that may interact to mediate intervention implementation and the response of recipients	
Organisation and infrastructure of homes	How care and service delivery is organised
	Type of home ownership
Culture and philosophy of the home	How leaders and managers create particular environments
	Orientation to learning
	How staff are valued
	Attitudes and approach to residents
	Relationships and connections between people
Macro context	Political factors—health policy, legislation
	Economic factors
	Societal, e.g. attitudes to older people
	Education systems
	Relationships with industry (continence products)
	Difference in systems across countries
Facilitation	Underpinning theories of action
Type A	• Quality improvement, organisational learning, and humanistic psychology—how individuals learn and apply that knowledge to improvement activities. • Within the PARIHS framework type A represents an approach to facilitation towards the left of the facilitation continuum [21].
Type B	• Critical social sciences, focussed on enlightenment, empowerment and emancipation—that enable individuals to develop new understandings about what needs to be changed and how to change it, including (1) understanding, (2) choosing and development appropriate strategies, (3) doing and (4) evaluation. • Within the PARIHS framework, type B represents an approach to facilitation towards the right of the facilitation continuum [21].
Internal–external facilitation	The chain of action between internal (IF) and external facilitators (EF)
Buddy	Relationship and dynamic between internal facilitator and buddy
Facilitator characteristics	Experience, knowledge and engagement of individual facilitators
Potential impacts	• Including anticipated and unanticipated, and reach and potential spread • Changes to continence practice - Improved assessment - Appropriate use of products - Revised continence local policy - Introduction of new practices and activities • Positive impact on residents’ and next of kin experiences • Positive impact on practitioners’ experiences, attitudes and learning • Positive impact on internal facilitators’ skills, confidence, experience, knowledge (*and* values with respect to type B) • Potentially positive impact on care home context (type B)

At this second workshop, a number of hypotheses which threaded together the ideas into theories were developed jointly by participants and study team, which are framed here as ‘if-then’ statements [22, 23] (Table 2).

**Table 2:** Initial theories expressed as ‘If-Then’ statements

• If home contexts (i.e. organisation, infrastructure, culture and philosophy, macro) align with the particular approaches to facilitation and their underpinning theories of action, and with facilitators’ characteristics, then this will prompt both anticipated and unanticipated effects on continence practice, residents, facilitators and homes.	
• If contextual conditions and characteristics of home staff, including home managers, are supportive, then this will prompt the enactment of the internal facilitator activities and practices proposed by the type A and type B programmes, including the following:	
o The interaction between facilitators, home managers and other informal leaders	
o May influence how successfully a facilitator can enact their role	
o The characteristics of leaders at various levels of the health/social care	
o Organisation will impact on implementation processes and outcomes	
o Implementation processes and practice changes will be hindered in organisations	
o Where there is limited ‘slack’ (time, space)	
o The degree of ‘fit’ between facilitation and facilitator characteristics	
o Organisation’s context and culture will impact implementation processes and outcomes	
o A home’s motivation to implement changes will influence the effect of facilitator activities	
o The nature and quality of the internal (IF)—external facilitator (EF) relationship, and the contents of the support programme (including support of a buddy) and the degree of ‘fit’ between internal facilitators and type of facilitation will prompt support and development that may have the potential to influence internal facilitator’s abilities, skills and knowledge to enact their role in practice, which could improve resident outcomes and experiences.	
o A potential for type B to have a greater effect because its holistic approach, longer intervention period and opportunities for more sustained support.	
• If research-based recommendations are introduced and integrated into the facilitation development programmes and into the homes, then this will prompt improved continence care processes, outcomes and resident and staff experiences.	

#### Data collection

Multiple qualitative methods were used to test these realist theories:

*Semi-structured interviews*: audio-recorded at baseline/pre-intervention, 6 (T1), 12 (T2), 18 (T3) and 24 (T4) months post the intervention initiation. Country-based research fellows undertook interviews in their native language using a consistent approach. Interviews were guided by a schedule that was developed from the realist theories and tailored to data collection time points. Key informants included site managers, nursing staff, facilitators, residents and next of kin, and relevant external stakeholders such as regional directors.

*Non-participant observation*s of health care and implementation activities were undertaken at least three times across data collection points in each intervention site using a consistent approach involving piloting the observation protocol. Data were recorded in field notes using Spradley’s nine dimensions of observation (space, actors, activities, objects, acts, events, time, goals and feelings) as a guide [24]. We focused on situations where residents were assisted with the management of urinary incontinence and implementation activities in each site. Observation of care necessitated an unobtrusive, sensitive approach and with consent.

*Site and country reports* were kept and included history and/or events affecting the care of older people: current demographics, legislation and political agenda; payment and organisation of nursing homes, staffing, resident turnover, and any new routines.

*Facilitator activity logs* completed by the IFs included activities, purpose, time spent, others involved, resources used, comments on what went well and what went less well.

The amount of data collected within each site depended on how conducive the home context was to data collection visits. This accounts for a variation in data collected (Table 3).

**Table 3:** Data collected

		Country				
		England (Eng)	The Netherlands (Neth)	Republic of Ireland (RoI)	Sweden (Swe)	Total
Data collection	Observations of care (hours)	38.25	68	84	142	333
	Facilitation activity Observations (hours)	0	4	21	14	39
	Staff interviews	60	55	234	76	357
	Resident interviews	29	49	43	31	152
	Next of kin/carer interviews	14	30	36	29	109
	Stakeholder interviews	18	27	20	55	128

#### Data analysis

Interview and observation data were transcribed in full and managed in Atlas Ti 6.2 and NVivo 9.

**Fig. 2:**
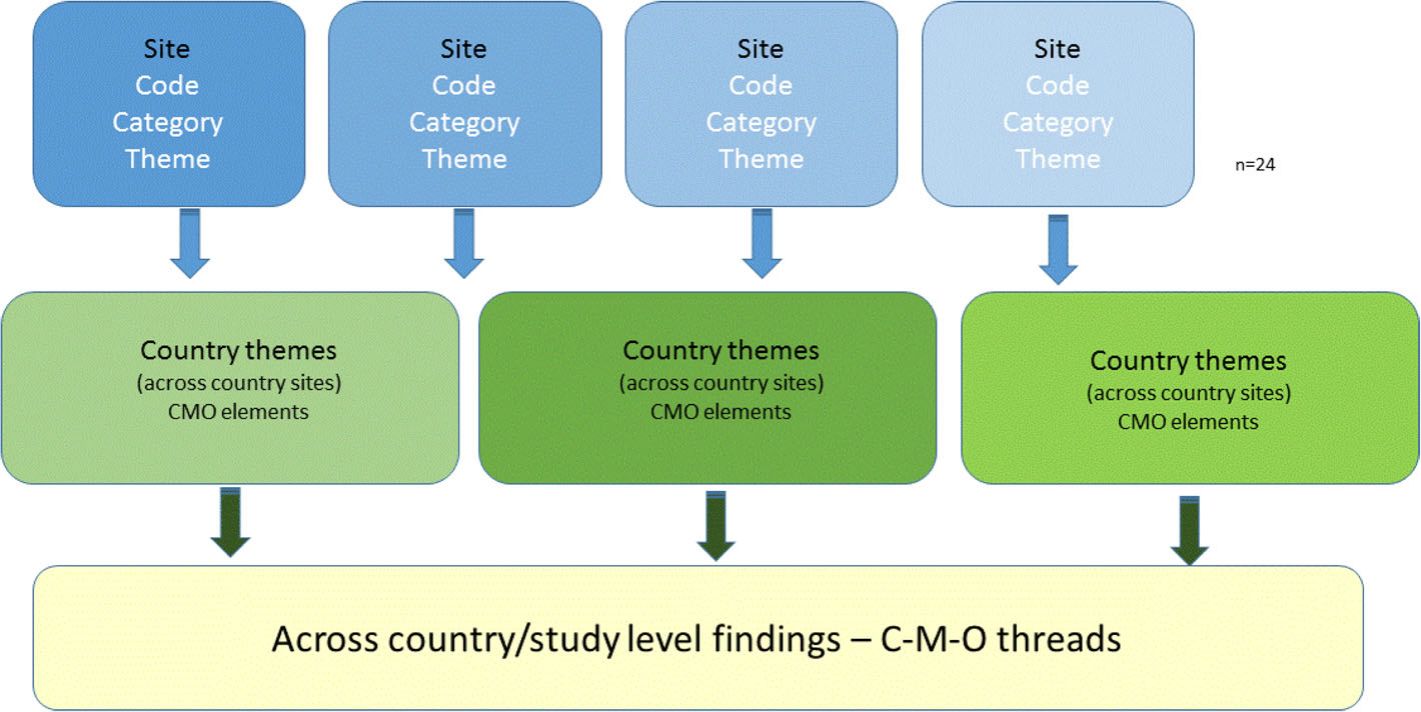
Analysis stages

A combined inductive and deductive content analysis approach was used. Data were first analysed within country, within site and within data set, per data collection point. Coding was undertaken within countries by country research teams (CM/CH, TN/TvdZ, PS/CM, ACE) to enable within country reliability checking. Country level coding was then shared at cross-country meetings, which involved a wider group of investigators (JRM, KS, GH, BMc). The starting point for analysis was the framework concepts (Tables 1 and 2). Sub-categories and categories that were developed from interview data were then used to analyse observation texts. Afterwards, sub-categories and categories were formed into themes, a process that was undertaken by country research fellows (CM/CH, TN/TvdZ, PS/CM, ACE) and country principal investigators (JRM, KC, BMc, LW). At this point, themes were translated to English including supporting quotations, for the purpose of country level, and then cross-country analysis (Fig. 2).

Cross-country analysis was managed through monthly teleconference and six monthly face to face meetings and began after the 6-month follow-up. These meetings involved research fellows (CM/CH, TN/TvdZ, PS/CM, ACE), country principal investigators (JRM, KS, KC, BMc, LW) and wider FIRE team members (GH, ALK, AT). Involving different investigators at each stage provided opportunities for challenge and cross checking of both analysis processes and emerging findings. At this stage, the development and refining of context-mechanism-outcome threads was undertaken. This involved searching for context, mechanism, outcome elements and patterns from across the themes through a deliberative and inductive process.

## Results

Findings from the trial showed no significant difference between study arms; all study arms improved on the primary outcome (documented compliance with continence recommendations) over time in all countries, but this was not statistically significant [18]. The 12-month type A and the 24-month type B facilitation interventions did not have different levels of impact on documented compliance with recommendations. Both facilitation groups showed significantly better documentation in three outcomes: cognitive impairment, depression and incontinence-associated dermatitis between baseline and 24 months, although these were based on small numbers [18].

Findings from the process evaluation are expressed as realist CMO configurations. Where we observed a difference between responses to type A and B facilitation, this is highlighted; however, findings surfaced similar issues irrespective of the type of facilitation approach.

### Aligning to needs and expectations

The content and approach of the two facilitation programmes (*context*) prompted variable opportunities to align and realign support (*mechanism*) with the needs and expectations of IFs and homes. This influenced their level of confidence in fulfilling the facilitator role, challenged an ability to deliver the intervention as planned, a compromise to intervention content exposure, and a continuum of engagement from sustained-partial-no engagement (*outcomes*).

The initial theories prompted us to examine issues of fit between, and in combination with, the type of facilitation programme, and the needs of the individuals and homes, and the nature of the support provided by the programme and external facilitators (EF). Findings show that alignment of these characteristics was important for the confidence of the IF to enact the facilitation role as intended, and therefore, the level of engagement there was in the programme in general. Factors that affected the fidelity of the intervention are summarised in Table 4.

**Table 4:** Fidelity to intervention

Factors	Type A facilitation	Type B facilitation
Variability in selection, preparation and drop out of IFs	• 7 of the 8 homes selected an IF to attend the 3-day residential programme. • 1 IF was selected later and completed a shorter development programme • 2 IFs withdrew shortly after the start of the intervention due to ill-health: 1 was replaced by a buddy (without training), and 1 was replaced by a nurse who did not meet all of the selection criteria, and who completed a shortened development programme.	• 6 of the 8 homes selected an IF to attend the 5-day residential programme (no IFs from one country attended). • 1 IF was recruited later and months later completed a condensed development programme. • Of the 6 IFs who participated in the full 5-day programme, 1 withdrew approximately 3 months after the start of the intervention due to ill-health and was replaced by a buddy who did not attend the initial programme or join the teleconferences; 1 other left for a new job and was replaced by someone who did not meet all the selection criteria. Whilst she attended a condensed development programme, she later withdrew from the project.
Variable engagement in the facilitation programme	• Following the residential programme 2 sites only engaged in a limited way. For example, one of the IFs had limited skills and access to IT making engaging in activities such as audit and feedback a challenge. • IFs from 2 sites participated in all 12 teleconference meetings; 2 sites in 3; attendance by IFs from the other 4 homes varied from 5 to 10 meetings.	• 1 site did not engage in the facilitation intervention and 1 other site in the same country disengaged soon after the start of the programme. • 18 monthly teleconference support meetings were held. 1 site participated in all 18 teleconferences. Attendance by the other sites varied between 10 and 15 meetings.
Progress according to plan	• None of the 8 homes were able to implement the plans devised at the residential programme, which included audit and feedback activity related to each of the guideline recommendations. • Partial implementation was achieved with 4 homes completing a baseline audit of the 4 recommendations and devised follow-up action plans • 4 homes addressed 2 or less of the recommendations	• 4 of the 8 homes created plans for developing more person-centred cultures. • 1 home made significant progress in advancing this plan and the others made variable progress. • Only 1 home was able to demonstrate progress in developing the quality of practice.

The responses to the type of facilitation, formed at the initial residential development programme, were important precursors to how well aligned and relevant the approach was perceived to be by the individual and to the home. The IF went through a process of sense making. Whilst a number of IFs expressed that they had been empowered by the residential experience and the enthusiasm of the EFs, there were differences in the IFs as to the extent they felt aligned with the facilitation approach, and theoretically, practically and emotionally equipped to enact the role. Additionally, whilst in both types of programmes, IFs were unsure about how they were going to translate what they learnt into practice, this perception appeared to be particularly evident in the numbers of accounts reported by those experiencing the Type B residential programme, for example:

Yes initially I thought, Jesus…with all these creative methods, where will this lead to, but I did experience it personally and how illuminating it was. Nevertheless I constantly wondered how am I going to do this on my unit with those persons... (IF type B, baseline, site 1 NL), and, after the residential, I was exhausted. For five days I just sat there, demolished, and like ‘where do I start.’ (IF, site 5, Swe, T1, type B).

Following the residential programme, support for the IFs switched to teleconferences, which whilst welcomed by most IFs, participants presented two challenges. The first, engaging in the group dynamics of a teleconference, including for most, in a language that was not their own:

The monthly teleconference meetings were very tiring because all was in English using telephone, so you do not see the others. We did not know the people either because we entered the project later…some people dominated the conversations…They had lots of questions…They had the advantage of the language (IF type A, 12 months, site 5, NL)

The second challenge was a feeling that there was a lack of opportunity to tailor support to their particular needs in real time, which meant they lacked confidence to act on advice that was provided in the monthly teleconferences:

...every time I heard [EF] it seemed logical, but the moment I got to the institution and had to translate it to actual practice I could not find any resemblance (IF type B, site 3, NL).

Consequently, facilitators felt unequipped to act out their facilitation role. This finding is also linked to the personal characteristics of the IF, described later, which mediated their ability to engage with the requirements of the role and programme.

Further, in relation to alignment of need and expectation, there had been a mismatch in some IF's and home manager’s perceptions about the facilitation programme. Misalignment related to the programmes’ intentions around development of people to be facilitators, versus the knowledge and tools required for putting best practice in place for continence care:

… you know we already use…the assessments...and the products…if it was going to be a case that you will be introducing new ways of doing things…but that’s not what it was about, so, no, I wouldn’t do that again (IF, type B, 12 month, Eng). In this example, the IF only attended one teleconference and then did not participate further in the programme.

As a result of all these factors, although the ‘dose’ of the intervention provided by the EFs within each programme was delivered, the resulting response and actions of the IFs were mixed; and thus, the potential of what they did to impact on practice was also variable.

### Prioritisation

The success of intervention implementation was largely dependent on whether sites prioritised their involvement in both the study and the facilitation programme. In contexts where interventions were timely coinciding with a regulatory requirement, and/or a need to improve continence care, and where there were fewer disruptions such as changes in staff and management (*context*), this prompted the prioritisation of the project (*mechanism*). This resulted in a release of resources (time, staff and material resources), and a more sustained commitment to the study and facilitation intervention (*outcomes*).

The initial realist theories prompted us to consider the implementation context and conditions that might enable or inhibit facilitator activities and role enactment. We found that there was a mutually reinforcing relationship between regulatory expectations (macro context) and home (meso context) managers’ motivation to prioritise continence and therefore engagement in the study. For example, in the Republic of Ireland, regular Health Information and Quality Authority inspections were used as an incentive to sustain engagement in the project. In Sweden, the prioritisation of urinary incontinence was reinforced in national guidance and by external agent’s expectations:

…we have a guideline (on UI) in the regulations, so whenever a resident moves into site x…they should be offered a basic UI assessment…it’s not negotiable…because you have started the [FIRE] project they now sense they really have to do something about it (Community Chief Nurse, Baseline, Swe).

There was also a reinforcing relationship between home managers and IFs’ ability to participate fully in the facilitation programme and in enacting their role. The dynamics between managers and facilitators were continually negotiated over the intervention implementation period. Where facilitators were given the authority through protected time to carry out activities, including attending monthly teleconference support meetings, this was a function of managers’ prioritisation of the project. As managers varied in their commitment to being involved, often because the day to day demands of running a home took over their attention, subsequent support was patchy or absent:

I did ask for protected time for a couple of the teleconferences but no cover was forthcoming (IF, T2, RoI, type A).

Conversely, there were examples in the data where managers had been able to consistently prioritise the project, which resulted in resources for the IF, particularly in terms of time to work as a project team:

…it’s been really good that we had had the time…we have had the time and energy to discuss things (nurse, T2, Swe, type B).

Money to enable backfill for IFs was available; however, difficulties in finding suitable replacements meant it was not always taken up.

Change in management and/or ownership of a home was generally disruptive to prioritising project related requirements, such transitions were a frequent feature of the implementation context in all of the countries. Losing the original sponsor of the study frequently delayed, and sometimes, completely curtailed activity. Additionally, frequently changing staff or team leaders made it difficult for IFs to sustain the project as a priority at a unit level:

I have openly declared to facilitators they cannot expect anything from the project at the moment. After summer I hope everything will settle again (Manager, T2, type B, NL).

This issue was particularly challenging in homes that were smaller (particularly the case in England), where there was a more limited flexibility in workforce deployment.

### Engagement in attitude and action

In contexts where the study was prioritised (including release of resources time, people, tools and infrastructure) and where managers and staff were supportive (*context*), this prompted collective engagement (as an attitude and action) with the facilitation interventions (*mechanism*) by managers, IFs and other staff. This resulted in IFs undertaking activities, which resulted in some practice changes (e.g. continence assessment) and impacts on attitudes and beliefs (*outcomes*).

As described earlier, the consequence of prioritisation was a commitment [or less so] to the project. This outcome forms the condition or context for greater engagement in both attitude and action with the facilitation interventions. The level of engagement reported and observed varied from withdrawal from programme activities (but not from the study), to patchy participation in the monthly support teleconferences, to some facilitators completing the programme. In this sense, engagement referred to both facilitators’ attitudes—‘I can’t do that’ (IF, T2, NL, Type B), as well as their actions—‘I dropped out of it [support programme]’ (IF, T2, Eng, type A).

Where there was not a supportive context (e.g. little support from colleagues, managers, and not enough resources such as backfill time), IFs struggled with the perceived costs of overcoming the challenges, and some gave up. Their ability to overcome these challenges was also inhibited by remote teleconference-based EF support. However, in contrast, in these situations some IFs had been encouraged to engage their local ‘buddy’ as a source of support:

…I do not think she’s fully comfortable, so *** has buddied up with her and is sort of the driving force behind it…they are spending time together and doing things (Manager, T1, Eng, Type A).

A more engaged IF tended to lead to more engaged home staff. In all facilitation intervention sites, there was some staff resistance to the practice changes needed to align with the guideline recommendations, such as continence assessment. However, active facilitators who engaged staff through meetings, team-related activities, workshops and role encouragement resulted in some success, including for example, the implementation of a new continence care screening and assessment form. This ability to engage home staff was facilitated by setting up a local project team in some sites, which became part of the support structure for the IF.

Whilst there was no impact on the primary outcome, interview and observation data showed that some facilitators had made changes to continence practices, such as introducing improved fluid monitoring, and in changing staff perceptions and approaches. Examples of making a difference to residents were also evident, for example:

…the nurse has investigated when I have to pee to see if we could do something about my incontinence. We did this together (Resident, T2, NL, type B).

There was also evidence that specific facilitation intervention activities had led to perceived changes in thinking, for example:

…the culture workshop had an even bigger impact than we expected, it was not about the collection of data alone, but an action in itself. It resulted in consciousness among staff about the impacts of incontinence for the client’ (IF, T3, months, RoI, Type B).

In contrast, data from follow-up interviews revealed that standard dissemination sites did not use the urinary continence recommendations or the implementation guide that they had received.

### Learning over time

IFs’ personal characteristics and abilities, including personal and formal authority, in combination with a supportive environment prompted by managers (*context)* triggered the potential for making sense and learning through the support programme over time (*mechanism*), which could result in a sense of confidence and personal growth, and enactment of the facilitation role (*outcomes*).

Whilst the starting point for most of the IFs was enthusiasm and an eagerness to succeed, their ability to carry out their role, including suggested facilitator activities, appeared to be linked to their level of authority to act, which was associated with credibility, confidence and perseverance when facing challenges. Despite a set of criteria for the selection of IFs, the practicalities of identifying someone who fitted all of them was a challenge with only 6 of the 16 sites recruited an IF who met the essential facilitation criteria and stayed in post for the duration of the study. This resulted in mixed cohorts of facilitators in each arm of the intervention, with authority to act being a significant factor in successfully enacting the role. Two forms of authority were evident: formal authority from their role within the home and/or delegated by the home manager to be an IF, and personal authority, which the IF engendered amongst those with whom they worked. The levels of authority varied amongst the IFs. When there were challenges, it was the resilience and persistence of the IFs, which in some cases was reinforced by encouragement and active participation from managers, which kept some momentum going.

As each facilitator progressed on their facilitation journey, we observed some critical junctures in their learning. There was a critical point immediately after the residential development programme at the beginning of the intervention period when the issue of alignment of IFs and home expectations, and not knowing ‘where to start’ was most evident. Facilitators’ ability to connect meaningfully in monthly calls were additional critical junctures, with some reporting challenges with understanding the language of facilitation and implementation (in addition to conversing in a foreign language) as described earlier.

However, over time, and with the teleconference support from EFs, and for some, the input of buddies, we observed a growing ability and confidence in some facilitators to act in accordance with the particular facilitation approaches. Additionally, whilst there were no significant differences in effectiveness between the interventions, there was increasing compliance with recommendations over time, suggesting improvements [18]. Key characteristics identified from field notes, interviews with managers and external and IFs that made some facilitators (irrespective of their allocation to type of facilitation or country setting) more successful than others are included in Table 5.

**Table 5:** Personal characteristics of more successful facilitators

Motivation to take on the role	
Desire to learn	
Years of nursing experience (because it helped with authority)	
Confidence in self and in working with others	
Eagerness to succeed	
Perseverance (particularly when things are hard going)	
Visible enthusiasm	
Commitment to improving the quality of care for older people	
Good communicator	

Data from facilitator activity logs, interviews and observations shows that learning and developing over time resulted in some facilitators enacting their roles through activities that made the particular facilitation approach they were aligned to more visible (see Table 6). Additionally, their learning pervaded other aspects of work life:

I suppose the big thing for me has been the personal journey…It goes into everything now not just continence, not just person centred care…It’s getting them to think for themselves…(IF, T3, RoI, type B).

**Table 6:** Activities related to facilitation type

	Underpinning theories	Activities evident of facilitation type
Type A	Quality improvement, organisational learning, and humanistic psychology—how individuals learn and apply that knowledge to improvement activities Within the PARIHS framework type A represents an approach to facilitation towards the left of the facilitation continuum (Harvey et al. 2002).	• Set up project group. • Developed action plans. • Developed posters and fliers about the project. • Audit—identify what needed to improve in continence practice. • Presentation of data in poster. • Development of information leaflets. • Development of new continence assessment forms. • Development of continence care plan. • Supported staff to complete the assessment forms.
Type B	Critical social sciences, focussed on enlightenment, empowerment and emancipation—that enable individuals to develop new understandings about what needs to be changed and how to change it, including (1) understanding, (2) choosing and development appropriate strategies, (3) doing and (4) evaluation. Within the PARIHS framework type B represents an approach to facilitation towards the right of the facilitation continuum (Harvey et al. 2002).	• Formed a project group of stakeholders. • Values clarification exercise. • Self-administered leadership questionnaires. • 360° feedback from colleagues. • Asked staff to complete Context Assessment Index. • Provision of person-centred care presentations to staff. • Interviewing residents with urinary continence. • Using stakeholder group to identify priorities, agree actions, evaluate progress. • Reviewed practice, revision of policies, including assessment forms.

### Summary

**Fig. 3:**
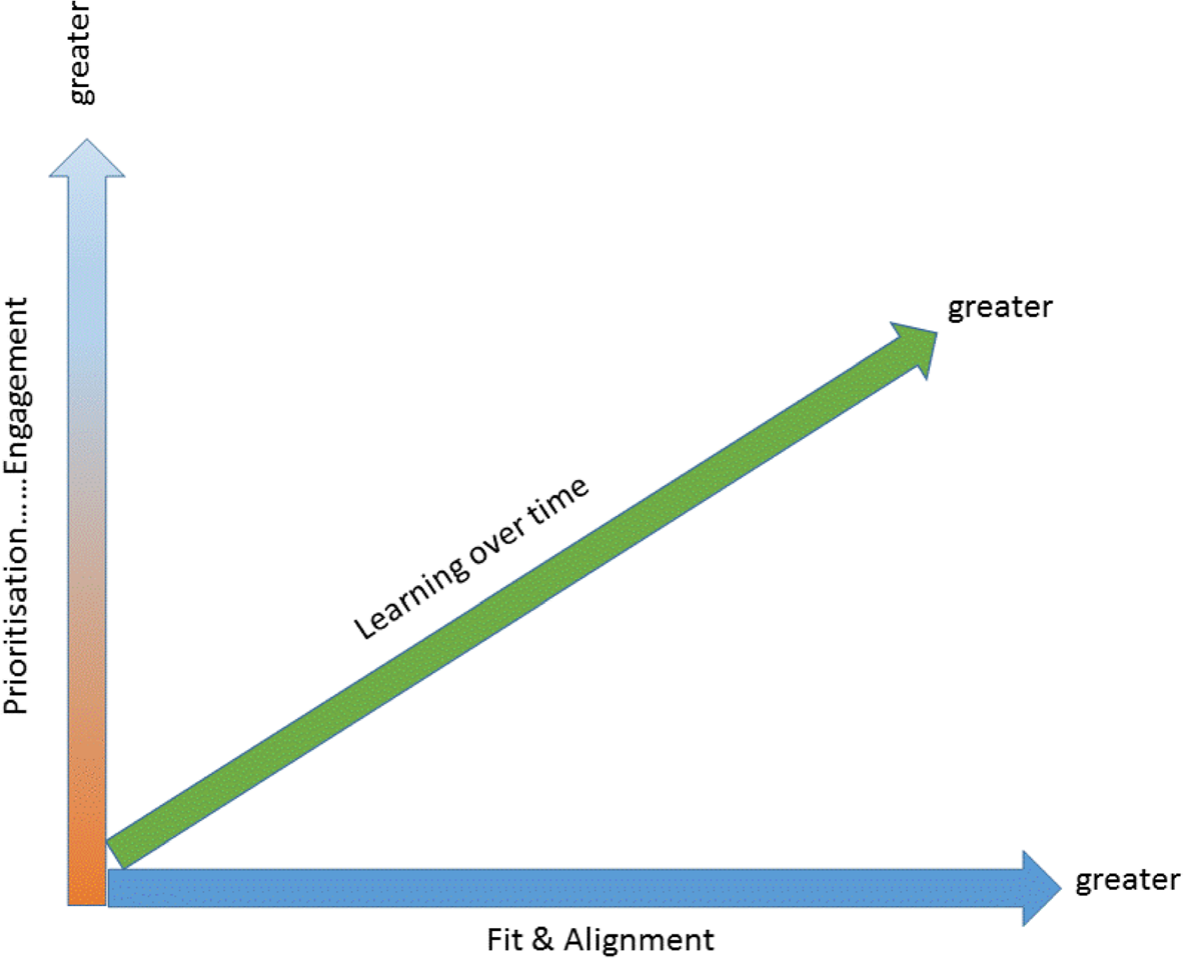
Mechanism activation continua

In summary, findings show there were a number of mechanism activation continua [19]. Figure 3 shows that the combination of greater activation of prioritisation and engagement, together with greater activation of fit and alignment of the intervention to the needs and expectations of IFs and homes, is linked to activation of learning over time. The impact of learning over time was in the activity undertaken relevant to the type of facilitation and, in some cases, to implementing practice changes.

## Discussion

In this realist-informed process evaluation, we have elucidated responses to facilitation intervention implementation within the context of an RCT where neither facilitation approach was effective in significantly affecting the primary outcome [18]. Process evaluation findings showed that there were some impacts to practice, but these were not distinguishable between the two facilitation types. This was unexpected, as type B facilitation was planned to be a more intensive and holistic approach over a longer intervention period and with more support from EFs than type A. We had theorised that this additionality might result in greater impact [5, 21]. However, in reality, both facilitation types experienced similar challenges in delivery, which meant that the fidelity and dose of intervention as standardised for the trial was diluted. As such, the intervention as theorised was not delivered as intended.

**Fig. 4:**
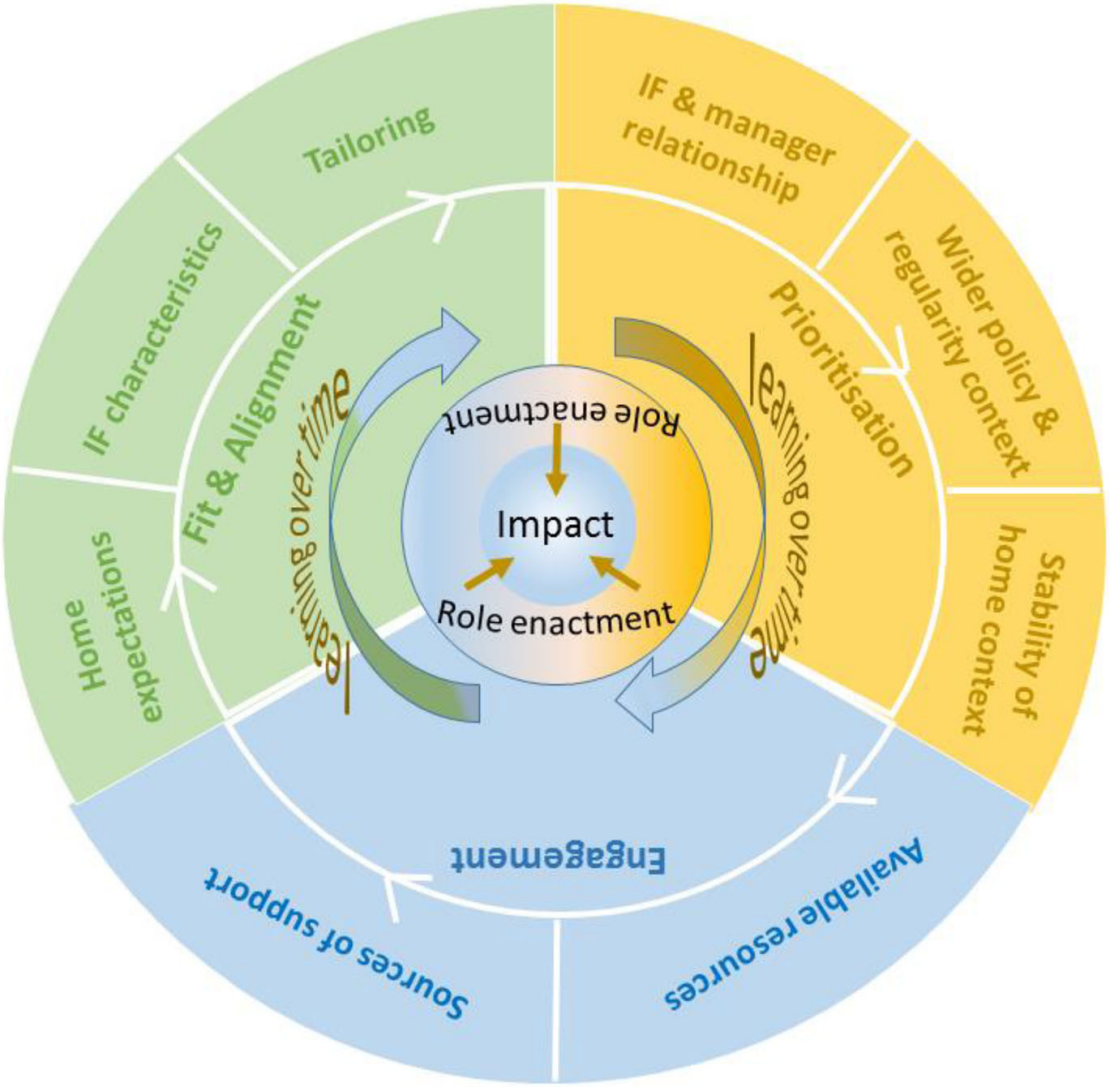
Representation of contingencies between CMOs

In realist terms, the CMOs explained how the resources and opportunities created by both facilitation interventions were taken up (or not) in different contexts. The interconnections between these CMOs are represented in Fig. 4.

The resultant framework illustrates that these elements worked in combination as a mid-range theory [10]. However, the impact of these combinations will be different depending on their arrangement within a particular circumstance, reflecting the significance of context to intervention implementation. Where there were impacts from being involved in the facilitation intervention, this was due to individuals enacting the facilitator role, which they achieved through learning over time. Learning over time and enacting the role was a function of a combination of elements. For example, prioritisation was important in that a reciprocal, supportive relationship between home managers and IFs combined with the stability of the home context, for example, staff complement and turnover. In turn, this combined with whether the project fitted with the priorities of the wider environment that home was operating within, for example, whether continence care was a particular focus for attention impacted on engagement. Prioritisation interacted with engagement, which was dependent upon the availability of the appropriate resources at the right time to enable facilitators to carry out their role, and whether they drew on the additional sources of support such as buddies when needed. Prioritisation, engagement, and fit and alignment together influenced how the facilitation interventions lined up with the expectations of homes and IFs, and the potential to tailor the approach including support structures to the on-going needs of both.

Whilst the elements in the framework have different combinations in different circumstances, we observed patterns. For example, one combination resulting in a positive response to the intervention related to a supportive reciprocal relationship between the IF and manager [25]. This reciprocity led to a release of resources in the form of time to engage with the programme, which was particularly evident in sites where the intent of the programme aligned well with both home and facilitators’ expectations. A different but consistent combination included a challenge to the response to the facilitation programme where the context of the home was disruptive. This was usually because of changes in managers, which resulted in a lack of stability, lack of buying in to the facilitation programmes and an inability to mobilise resources to engage fully, which left facilitators isolated.

The role of leaders and managers alongside facilitators, and as facilitators of implementation efforts themselves, is highlighted as a key ingredient for success by others [26, 27]. Specifically, the active and visible participation of managers in implementation interventions and processes is important for the allocation of resources and provision of support. For this study, early managerial buy in and engagement with the study itself was an obvious antecedent to supporting what was required to implement the facilitation interventions over a sustained period.

Other studies of facilitation have shown that it can take some time to affect outcomes [28]. In this study, we found that irrespective of facilitation type, for some IFs, there had been learning over time. This occurred where there was greater fit and alignment of the interventions to expectations, prioritisation and engagement, which had begun to result in some positive changes in practice. Learning over time was a feature within a rehabilitation research context in which an occupational therapist adjusted the way they worked with care homes and residents as they trialled a complex intervention and became more confident and proficient over time [16]. The idea of learning over time also fits with a realist logic of programme implementation, where we would expect to observe a dynamic interplay between the intervention, actors, contexts and mechanisms as the resources and opportunities created by the intervention are taken up, or not.

The realist process evaluation also highlights a challenge related to the delivery of an intervention like facilitation within the context of a randomised controlled trial. Any implementation effort requires work [29], including tailoring to local need [4, 30], which raises a question about fidelity versus adaptation of an implementation intervention such as facilitation. The manualised facilitation interventions in this study left little scope to particularise support to the individual needs and circumstances of facilitators as they changed over time, therefore, for example, where there were critical junctures or moments of crisis [31] for individual facilitators which could not be responded to and opportunities to support them lost. Inevitably, this affected facilitators’ confidence and expertise to enact the role. Reframing the idea of fidelity away from adherence to delivery of specific intervention components towards alignment with intervention function and process [32]; as a ‘thread that pulls together implementation processes within a trial along with the theories embedded in a complex intervention’ ([16], p446) may be more helpful. Arguably, this view provides a more flexible framework for assessing fidelity, including being able to contextualise interventions to the needs of specific circumstances whilst still being faithful to their underpinning theory/ies.

### Realist-informed process evaluation—strengths and limitations

**Fig. 5:**
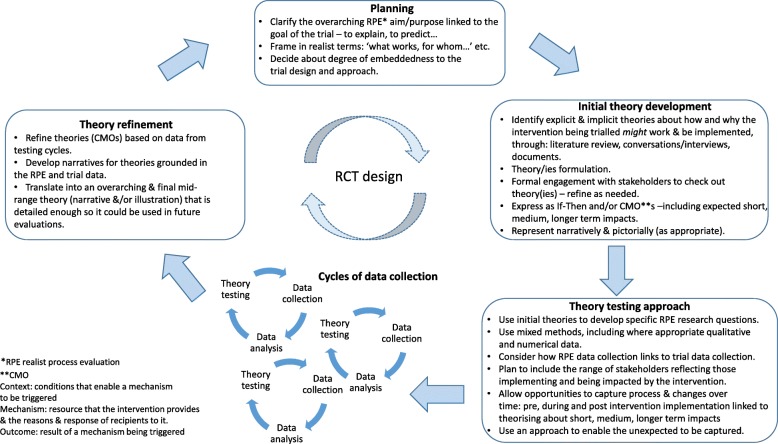
Realist process evaluation framework

Very few published examples of completed realist-informed process evaluations exist, and none at the scale of this study set in multiple country contexts. Indeed, much of the debate about combining realist inquiry with trials put these approaches in opposition [13, 14]. This presented a challenge because there was no example to follow, but an opportunity to fill a gap and contribute to the evidence base about realist inquiry alongside randomised controlled trials. Arguably, the strength of a realist-informed process evaluation is in the potential to provide greater explanatory power than potentially reductionist approaches centred on logic models. Whilst logic models are helpful for specifying intervention components as inputs and outputs, they can be less useful for developing contingent explanations between them. In this study, we have been able to provide an explanatory account of the antecedents and contingencies that account for the response to the resources and opportunities (i.e. realist mechanisms) offered by the facilitation intervention, moving beyond a list of facilitators and barriers and a conceptualisation of context as something that is static. As well as providing a richer explanation, the results should also be of more use to others embarking on research about facilitation because they provide an initial conceptual platform for further investigation [10]. Additionally, we offer a framework that identifies some co-ordinates and questions for realist process evaluations within randomised controlled pragmatic trials, which may be a useful starting point for others in future research (Fig. 5). The framework is based on our experience in this study and previous realist evaluation research projects conducted by some of the authors [33, 34], and some of the principles of conducting process evaluations described by Moore et al. [6, 7].

The strength of this study is that we drew on multiple methods to test and refine the programme theories through the project and included observations and activity logs. Data collected from these approaches complemented data from interviews, enabling a more trustworthy picture to emerge. It was also a strength to engage with stakeholders to develop our initial programme theory ideas and share findings as we progressed. Furthermore, a judgement about the credibility of the findings of this realist inquiry study can be verified if read alongside the other publications arising from this study [18, 35].

A large amount of data were collected, which through the data management and analysis process may have lost some of its site and country nuance, particularly as the last part of the analysis process was managed with data translated into English. However, our analysis process involving investigators at different stages also presented multiple opportunities to enhance the reliability of the resultant findings.

## Conclusion

This was a pioneering and complex study due to its scale and four-country context, which provided a novel circumstance in which to conduct one of the few realist-informed process evaluation as part of a randomised controlled implementation research trial. The CMO configurations were translated into a mid-range theory framework, which provides an explanation about the response to the facilitation interventions we observed in this realist inquiry. This shows that elements of fit and alignment, prioritisation and engagement can work together to determine a facilitator’s opportunity to learn over time, enact their role and have an impact, which could provide a useful conceptual platform for future facilitation research. In addition to providing a worked example, we have also outlined a realist-informed process evaluation framework that might be useful for future research of this nature as this approach continues to be trialled and developed.
